# Comparison of in-house and commercial real time-PCR based carbapenemase gene detection methods in *Enterobacteriaceae* and non-fermenting gram-negative bacterial isolates

**DOI:** 10.1186/s12941-017-0223-z

**Published:** 2017-07-10

**Authors:** M. Smiljanic, M. Kaase, P. Ahmad-Nejad, B. Ghebremedhin

**Affiliations:** 1 Center for Clinical and Translational Research, Institute for Medical Laboratory Diagnostics, HELIOS University Clinic Wuppertal; Witten/Herdecke University, Heusnerstr. 40, 42283 Wuppertal, Germany; 2 Department of Medical Microbiology, Ruhr-University Bochum, Bochum, Germany; 3 Department of Infection Control, University Medical Center Göttingen, Göttingen, Germany

**Keywords:** RT-PCR, Carbapenemases, *Acinetobacter baumannii*, MDR gram-negative bacteria

## Abstract

**Background:**

Carbapenemase-producing gram-negative bacteria are increasing globally and have been associated with outbreaks in hospital settings. Thus, the accurate detection of these bacteria in infections is mandatory for administering the adequate therapy and infection control measures. This study aimed to establish and evaluate a multiplex real-time PCR assay for the simultaneous detection of carbapenemase gene variants in gram-negative rods and to compare the performance with a commercial RT-PCR assay (Check-Direct CPE).

**Methods:**

116 carbapenem-resistant *Enterobacteriaceae*, *Pseudomonas aeruginosa* and *Acinetobacter baumannii* isolates were genotyped for carbapenemase genes by PCR and sequencing. The defined isolates were used for the validation of the in-house RT-PCR by use of designed primer pairs and probes.

**Results:**

Among the carbapenem-resistant isolates the genes *bla*_KPC_, *bla*_VIM_, *bla*_NDM_ or *bla*_OXA_ were detected. Both RT-PCR assays detected all *bla*_KPC_, *bla*_VIM_ and *bla*_NDM_ in the isolates. The in-house RT-PCR detected 53 of 67 (79.0%) whereas the commercial assay detected only 29 (43.3%) of the OXA genes. The in-house sufficiently distinguished the most prevalent OXA types (23-like and 48-like) in the melting curve analysis and direct detection of the genes from positive blood culture vials.

**Conclusion:**

The Check-Direct CPE and the in-house RT-PCR assay detected the carbapenem resistance from solid culture isolates. Moreover, the in-house assay enabled the identification of carbapenemase genes directly from positive blood-culture vials. However, we observed insufficient detection of various OXA genes in both assays. Nevertheless, the in-house RT-PCR detected the majority of the OXA type genes in *Enterobacteriaceae* and *A. baumannii*.

## Background

An increase of infection triggered by multidrug-resistant gram-negative bacteria is a global problem because it is related to longer hospitalization, increased morbidity and mortality rates and to increased medical costs. The production of β-lactamase enzymes is the most common mechanism of bacterial resistance [1]. Carbapenemases are β-lactamases that have the ability to hydrolyse all β-lactam antibiotics, including carbapenem [2]. Rapid diagnostic tests for the identification and detection of specific resistance mechanism are necessary for therapy and prevent dissemination. Different techniques can be applied to identify carbapenem-resistant gram-negative bacteria. Phenotypic tests are used for detecting the carbapenemase activity while molecular based assays identify the resistance genes [3]. Carbapenem-resistance can be detected by disc diffusion tests or Etest with imipenem, ertapenem or meropenem using agar plates or by automated systems (e.g. BD Phoenix, VITEK 2), selective chromogenic media for carbapenemase screening or the modified Hodge test (MHT) as additional phenotypic method [4]. The MHT is the only Clinical and Laboratory Standards Institute (CLSI)—recommended carbapenemase-screening method [4]. However, the gold standard for the detection of carbapenem-resistance are molecular based methods e.g. polymerase chain reaction (PCR)—either single- or multiplex PCR followed by a sequencing step if needed [3, 4]. However, these molecular methods are mainly implemented in reference laboratories. These diagnostic tools are necessary to administer the adequate therapeutic management for instance in septicaemia caused by carbapenemase-producing pathogens. Therefore, the distinction between carbapenem-resistance mediated by carbapenemases and those by other mechanisms is important for infection control in hospital setting. As the carbapenemase-producing gram-negative bacteria causing severe infections are generally multidrug-resistant, treatment options are compromised. Thus, the timely detection and monitoring of carbapenemases is important for therapeutic and prognostic means. Various carbapenemase genes may disseminate differently in the European countries, in the USA or the Asian countries. In the study region the predominance of the class D carbapenemase genes in *Enterobacteriaceae* and *Acinetobacter baumannii* is true [5].

In this study we established and evaluated a multiplex RT-PCR assay for the simultaneous detection of carbapenem resistance genes in gram-negative rods using the RT BD MAX™ system and compared the in-house RT-PCR with the commercial assay, the Check-Direct CPE kit.

## Methods

### Culture methods

#### Bacterial isolates

116 multidrug-resistant gram-negative isolates were collected from urine, blood-culture, wound swabs, tracheal secretions and other clinical specimens. Patient material were plated on Mac Conkey agar (BD, Becton–Dickinson and company, Heidelberg) and incubated at 37 °C over night. Species identification of these bacterial isolates was confirmed using MALDI-TOF mass-spectrometer. Carbapenem-resistance was approved using phenotypically methods (antibiotic disk diffusion and Etests on Muller–Hinton agar with imipenem, meropenem and ertapenem (BD Heidelberg, 10 µg and BD Phoenix system). All isolates have been characterized by the National Reference Centre (NRZ) at the molecular level.

For the identification of carbapenemase-producers directly from positive blood-cultures, negative Bactec™ Plus Aerobic/F blood-culture bottles containing clinical blood samples were inoculated with carbapenemase-producing isolates and incubated in a shaking incubator at 37 °C. For this purpose, a bacterial cell suspension was prepared with NaCl until a density of 0.5 McFarland turbidity standard was achieved. The blood-culture was inoculated with 500 µL of the bacterial suspension.

As negative controls we analysed 15 *Pseudomonas aeruginosa* and 20 *A. baumannii* isolates which were resistant to carbapenems but negative for the carbapenemase genes.

### Molecular methods

#### DNA extraction

Some colonies of an overnight bacterial culture were suspended in 1.5 mL of 0.9% NaCl and centrifuged at 13,000 rpm for 10 min. The supernatant was discarded, the pellet resuspended in 500 µL NaCl and incubated for 15 min at 95 °C. Thereafter the suspension was incubated for further 10 min at room temperature and centrifuged at 13,000 rpm for 10 min. The supernatant was transferred in a new tube and the DNA concentration was measured.

For the bacterial extraction from positive blood culture vials 1.5 mL of the blood-culture was centrifuged for 5 min at 900 rpm. 500 µL of the supernatant was transferred in a new tube, mixed with 500 µL SDS (2%) and centrifuged at 13,000 rpm for 2 min. The supernatant was discarded and the DNA was extracted from the bacterial pellet as described above.

### BD MAX™ diagnostic system

The BD MAX™ diagnostic system is an open RT-PCR system which allows the application of user defined protocols (UDPs). The advantage of this technology is the combined automatization of sample lysis, extraction, amplification and detection. This diagnostic system is time saving and avoids human errors. The fluorescence detection of the BD MAX™ is based on LEDs and photodiode filtering components to monitor fluorescence up to five different channels.

### Primer and probes design/sets for the detection of β-lactamase genes

The primers used in this study were designed after aligning variations of the target gene using MUSCLE (http://www.ebi.ac.uk/Tools/msa/muscle/). The used sequences were downloaded from the National Center for Biotechnology Information (NCBI).

The aligned sequence was then used to design primers with the NCBI primer designing tool (http://www.ncbi.nlm.nih.gov/tools/primer-blast) and ordered at Eurofins Genomics, Ebersberg. Initially, singleplex PCR using the mastercycler (Eppendorf, Hamburg) was done to proof the primer pairs. The probes for each designed primer were ordered at TIB MOLBIOL Syntheselabor. The primers and the corresponding probes are listed in the Table 1.

**Table 1:** Primer and probe sequences for the in-house RT-PCR assay

Gene	Primer pairs	Product size, bp	Probe
*bla*_KPC_	for-GCGATACCACGTTCCGTCTG rev-CGGTCGTGTTTCCCTTTAGC	183	6FAM-AGCGGCAGCAGTTTGTTGATTG–BBQ
*bla*_NDM_	for-TTTGGCGATCTGGTTTTCCG rev-ATCAAACCGTTGGAAGCGAC	101	LC610-AGACATTCGGTGCGAGCTGGC–BBQ
*bla*_OXA-23-like_	for-CGCGACGTATCGGTCTTGAT rev-CGCGACGTATCGGTCTTGAT	162	HEX-ACGTATTGGTTTCGGTAATGCTGA–BBQ
*bla*_OXA-48-like_	for-GGCACGTATGAGCAAGATGC rev-GTTTGACAATACGCTGGCTGC	182	^a^
*bla*_VIM_	for-CCGAGTGGTGAGTATCCGAC rev-GAATGCGTGGGAATCTCGTTC	337	Cy5-CGCTGTATCAATCAAAAGCAACTCATCA–BBQ

^a^The melting-curve analysis for the OXA gene variants was performed with the OXA-48 primer. For the multiplex RT-PCR analysis a Light Mix Modular from TIB MOLBIOL Syntheselabor GmbH Berlin was used for OXA-48

### In-house RT PCR on BD MAX™ system

For amplification, we used the Absolute qPCR Mix, no ROX (2×) (Thermo Fisher Scientific) and suspended the mix with 0.5 µL of the OXA-23 primer, OXA-48 Light Mix Modular, 0.75 µL of KPC, NDM and VIM primers, 0.3 µL OXA-23 probe and with 0.45 µL KPC, NDM and VIM probes. The primers primer master stock concentration was 10 pmol/µL for VIM, KPC, OXA-23 and NDM, and the probe concentration was 0.5 pmol/µL. 0.5 µL of the extracted DNA with a concentration of 80 ng/µL was added and a final volume of 12.5 µL were pipet into the microfluidic PCR cartridge (BD, Heidelberg).

The PCR was run at 95 °C for 5 min for 45 PCR cycles (95 °C, 15 s; 59 °C, 30 s; 72 °C, 11.7 s) followed by melt curve analysis for OXA-23 and OXA-48 variants with SYBR green from 40 to 100 °C in 0.2 °C steps. For the amplification and the analysis of the results the BD MAX software 4.32 A was used.

### Check Direct CPE on BD MAX™ system

Bacterial isolates were directly analysed from culture media. Therefore a bacterial cell suspension to an optical density of 0.5 McFarland was diluted 1:100 and approximately 500 µL of this suspension was transferred into the DNA sample buffer tube SB-1. The rack and instrument were prepared according to the manufacturers’ guidelines as well as the amplification program and the interpretation of the results.

#### Kappa (κ) statistics

To rather analyse the concordance between the two RT-PCR methods (in-house and commercial) for the detection of the various carbapenemase genes we performed kappa statistical analysis by use of the VassarStats platform http://vassarstats.net/index.html.

## Results

### Detection of the carbapenemase genes from solid culture isolates by use of the in-house method

A total of 18 KPC (*bla*_KPC-2_ and *bla*_KPC-3_), 19 NDM (*bla*_NDM-1/6, -2, -3, -5_ and _-9_) and 13 VIM (*bla*_VIM-1, -2, -4_ and _-11_) were detected by the in-house RT-PCR. Sixty seven (56.3%) of the 116 carbapenem-resistant gram-negative isolates harboured carbapenemase genes of oxacillinase-type carbapenemase (OTC)-variants (*bla*_OXA-23, -40, -48_, and _-58_-like). Within this family we observed the highest diversity of subtypes. The prevalence rate of the gene variant *bla*_OXA-23-like_ was 41.8% among the different carbapenemase genes, primarily in *A. baumannii* isolates. 57 (85.1%) of the OXA types were detected by multiplex RT-PCR by use of OXA-23/-48 primer pairs. The *bla*_OXA-23-like_ variants were amplified with the OXA-23 primer pair and *bla*_OXA-48, -162, -204_, and *bla*_OXA-232_ with the OXA-48 primer pair. Weak signals were observed during the amplification of *bla*_OXA-181_ (OXA-48-like) (*n* = 4 out of 5; 80%) with the OXA-48 primer pair. The remaining *A. baumannii* isolates and other gram-negative species carrying the genes *bla*_OXA-40_-like and *bla*_OXA-58_-like could not be detected by the in-house RT-PCR assay.

**Fig. 1:**
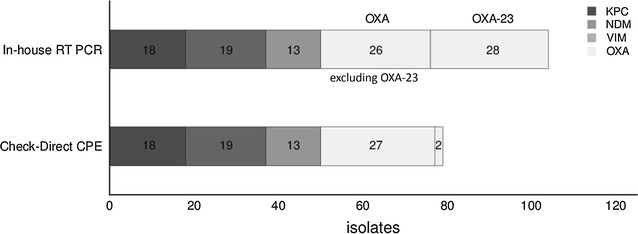
Comparison of the in-house PCR assay and the Check-Direct CPE assay for the identification of carbapenemase-producing isolates (*n* = 116 isolates)

In comparison to the in-house RT-PCR assay the isolates were analysed by use of the commercial Check-Direct CPE kit on the BD MAX™ system as well. All isolates harbouring the *bla*_KPC_, *bla*_NDM_ and *bla*_VIM_ genes were sufficiently detected by the commercial assay. One *bla*_NDM_ lost the resistance gene and has been excluded from the analysis. However, not all *bla*_OXA_ gene variants were detected by use of the Check-Direct CPE assay. Only two of the 28 *bla*_OXA-23_-like and all the *bla*_OXA-48_-like (including 16 OXA 48, one OXA-162, five OXA-181, one OXA-204, and two OXA-232) isolates were detected in the OXA-48-like channel of the BD MAX system. Moreover, the commercial system also detected incorrectly two of the 6 *bla*_OXA-72_ (OXA-40-like) isolates in the same channel. The two *A. baumannii* isolates harbouring *bla*_OXA-23_-like and *bla*_NDM-1/-6_ were only identified as NDM gene carrying isolates. Figure 1 illustrates the differences of the two assays in distinguishing the carbapenemase genes. The unweighted kappa (κ) for the KPC, NDM and VIM detection was 1—showing 100% concordance between the commercial and the in-house method—whereas for the OXA gene detection the unweighted κ was 0.4298 (95% CI 0.1251–0.7345). Noteworthy, both the in-house and the commercial RT-PCR assay did not detect any carbapenemase genes in our negative controls: 35 carbapenem non-susceptible *P. aeruginosa* and *A. baumannii* isolates. Therefore, the unweighted κ for the analysis of the non-carbapenmase isolates was 1.

**Fig. 2:**
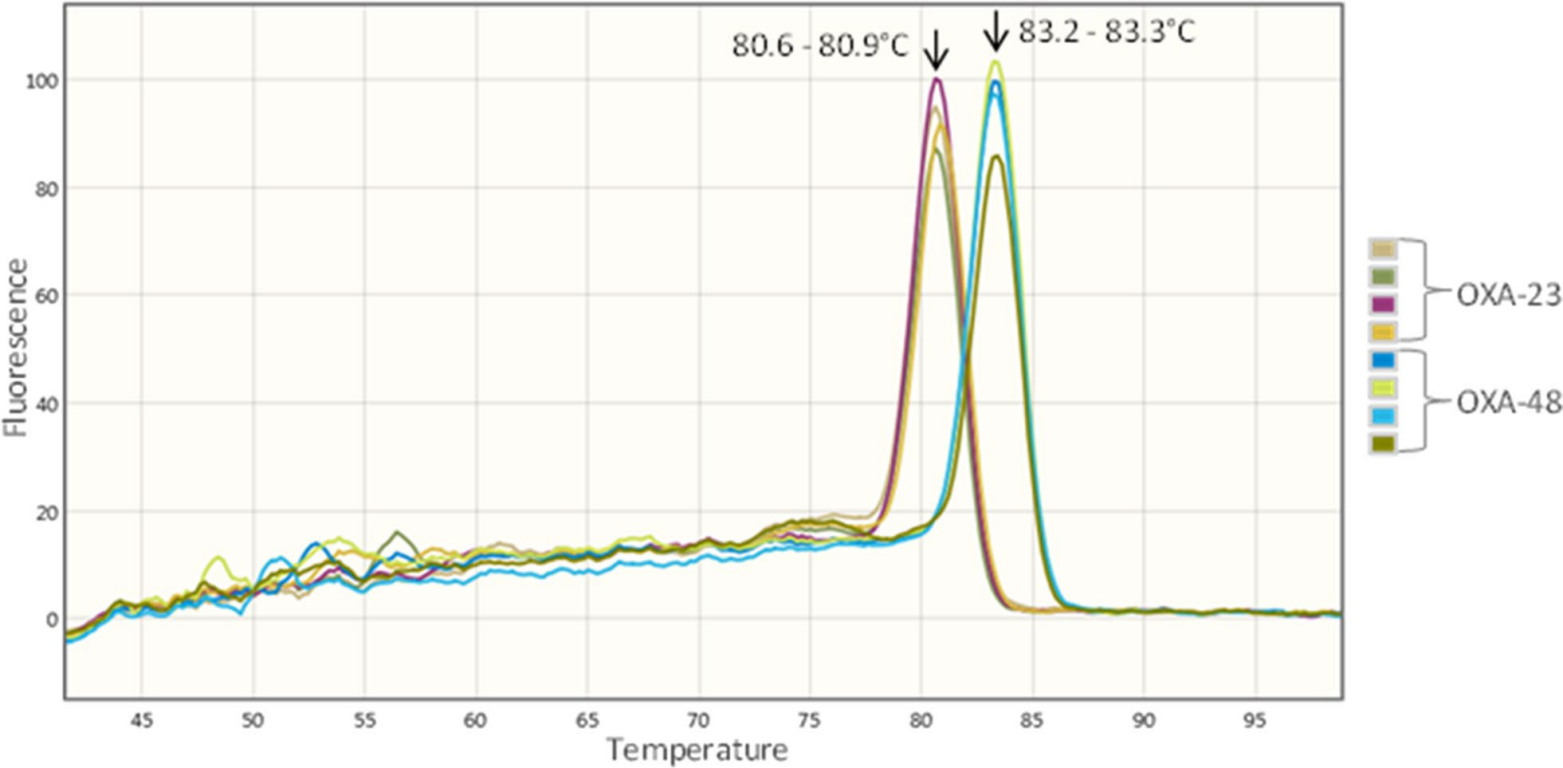
Melting-curve analysis of OXA-23-like and OXA-48-like gene variants. The carbapenemase genes were amplified by SYBR green multiplex PCR mix with OXA-23-like and OXA-48-like primer followed by melt curve analysis. The melting-curve was visualised at the wavelength channel of 475/520 nm. The melting peak for OXA-23-like was observed between 80.6 and 80.9 °C and for OXA-48-like between 83.2 and 83.3 °C

Due to the high prevalence rate of *bla*_OXA-23_ and *bla*_OXA-48_ variants a stepwise diagnostic workflow by use of melting-curve analysis on BD MAX™ system was performed to rather differentiate these OXA subtypes. Figure 2 demonstrates the results of the in-house assay for OXA-23- and OXA-48-like with distinct melting points (T_m_).

### Detection of the carbapenmase genes from positive blood culture

Based on the in-house RT-PCR assay, 58 of 116 investigated carbapenemase-producing isolates were additionally analysed directly from spiked blood-culture vials and all the *bla*_NDM_ and *bla*_VIM_ isolates were sufficiently detected. One (7.1%) of the 14 *bla*_KPC_ carrying isolates and five (19.2%) of the 26 *bla*_OXA_ gene variants carrying isolates were not verified by the in-house assay. The amplification of one *bla*_OXA-232_ (OXA-48-like) indicated weak signal and isolates carrying *bla*_OXA-181_ (OXA-48-like) were not detected directly from positive blood-culture vials. The two *A. baumannii* isolates co-harbouring the genes *bla*_OXA-23-like_ and *bla*_NDM-1/-6_ were detected in both channels (Table 2).

**Table 2:** Detection of carbapenemase genes directly from positive blood culture vials by use of the in-house multiplex RT-PCR (*n* = 58)

Species	OXA23-/48-like (*n* = 26)	NDM (*n* = 11)	VIM (*n* = 9)	KPC (*n* = 14)
*A. baumannii*	18^a^	2^a^		
*C. freundii*	1		1	
*E. asburiae*		1		
*E. cloacae*			2	
*E. coli*	2	4		2
*K. oxytoca*			1	1
*K. pneumoniae*	5	4	1	11
*P. aeruginosa*			4	
Detected by in-house RT-PCR	21	11	9	13

^a^2 isolates carrying *bla*_OXA-23-like_ and *bla*_NDM-1/-6_

## Discussion

Multidrug resistance in gram-negative bacterial strains is increasing globally and turn out to be a very urgent challenge in health care facilities. Several phenotypic screening methods have been developed to detect or confirm carbapenemase production. These phenotypic methods are time consuming and might be difficult to interpret, e.g. occurrence of extended-spectrum betalactamases (ESBL) and porin loss or AmpC production. Therefore, the resistance gene detection by specific nucleic acid detection methods is the gold standard [3, 4, 6].

In this study we established and evaluated a multiplex RT-PCR assay in the PCR-only mode on the BD MAX™ system for the simultaneously detection of carbapenemase gene variants and the data were compared to those gained by commercial test kit. Another advantage is the combined sample lysis, extraction, amplification and detection using the fully automated BD MAX™ system. Hindiyeh et al. [7] detected *bla*_KPC_ genes and Naas et al. [8] *bla*_NDM-1_ using RT-PCR. However, these assays can only detect the presence of one carbapenemase gene. Hofko et al. [9] reported the detection of carbapenemase gene variants with a multiplex SYBR green RT-PCR assay on the BD MAX™ system using two different master mixes (master mix 1: IMP-1, IMP-2, GES, VIM-2, KPC and 16S rRNA; master mix 2: OXA-23-like, OXA-48-like, VIM-1 and NDM). Nevertheless, this assay detects the carbapenemase genes in one channel (wavelength 475/520). The melting-curve analysis was performed to rather differentiate the different genes. In our RT-PCR assay we identified four different carbapenemase genes (*bla*_KPC_, *bla*_NDM_, *bla*_OXA_ and *bla*_VIM_) in four different channels using five different probes labelled with four fluorescent dyes for each primer pair. Based on the in-house PCR assay all investigated isolates carrying *bla*_KPC-2, -3_ (18/18), *bla*_NDM-1/-6, -2, -3, -5, -9_ (19/19) and *bla*_VIM-1, -2, -4, -11_ (12/12) gene variants were correctly identified. In case of the two bacterial isolates carrying more than one carbapenem-resistance gene (*bla*_OXA-23_-like and *bla*_NDM-1/-6_) both variants were identified simultaneously.

The detection of oxacillinases seems to be more challenging. Today five main phylogenetic subgroups of the OXA-type carbapenemases (OTC) have been recognised in *A. baumannii*: OXA-23-like, OXA-40-like, OXA-51-like, OXA-58-like and OXA-143-like. The master mix containing two different OXA primer pairs (OXA-23- and OXA-48-like) detected 53 of 67 (79.0%) *bla*_OXA_ genes. The in-house assay was specific in a manner that it did not detect *bla*_OXA-40_- and *bla*_OXA_-_58_-like variants. However, the detection of *bla*_OXA-181_ (4 out of *n* = 5; OXA-48-like) in *Klebsiella pneumoniae* isolate was insufficient. Further primer pairs aligned to the undetected genes could be included to rather enable the detection of more OTC variants. This was also consistent with the commercial Check-Direct CPE kit which also missed some oxacillinases positive isolates. The kit identified all five investigated *bla*_OXA-181_. Unfortunately, this kit was not specific by detecting two of the six *bla*_OXA-40_-like isolates. The primers should not cover this enzyme sub-group. However, the identification of *bla*_OXA-23_-like variant remains as one of the challenging issues that yet to be improved. Only two (7.1%) of the 28 *A. baumannii* isolates carrying this gene variant were identified with the Check-Direct CPE kit. Lau et al., Antonelli et al., and Nijhuis et al. [10–12] reported 100% sensitivity for the detection of carbapenemase-producing bacteria comprising of *bla*_NDM_, *bla*_KPC_, *bla*_VIM_ and *bla*_OXA-48_-like performed by the Check-Direct CPE kit which is consistent with our results. The comparison of the simultaneous detection of *bla*_OXA-23_-like gene variants and the other carbapenemase genes with previous studies was not possible due to the fact that no corresponding data exist yet. However, in comparison to the Rotor-Gene instrument Monteiro et al. [13] reported the detection of *bla*_IMP_, *bla*_OXA-48_-like, *bla*_NDM-1_, *bla*_GES_, *bla*_VIM_ and *bla*_KPC_ in 21 *Enterobacteriaceae*, 1 *A. baumannii* and 8 *P. aeruginosa* isolates (*n* = 30) using the Rotor-Gene 6000 instrument with 100% sensitivity. Compared to the BD MAX™ where the genes were differentiated in five separate channels by use of the Rotor gene instrument, the genes were distinguished via melting curve analysis. Ellington et al. [14] reported in 2016 100% sensitivity for the detection of *bla*_KPC_, *bla*_NDM_, *bla*_OXA-48_-like and *bla*_VIM_ carbapenemase genes using a multiplex real-time PCR assay on the Rotor-Gene Q as well as on the ABI 7500 instrument. The ABI Prism 7500 Fast instrument was also used by another research group to simultaneously detect *bla*_NDM_ and *bla*_KPC_ genes [15]. Yang and Rui [16] reported the simultaneous detection of *bla*_NDM_, *bla*_OXA-23_-like, *bla*_OXA-40_-like, and *bla*_OXA-58_-like genes in *A. baumannii* and other *Acinetobacter* spp. by performing two multiplex RT-PCR assays on the ABI Prism 7500 FAST apparatus. However, their assays did not include the identification of different carbapenemase gene variants from other species, so that the comparison of the simultaneous detection of *bla*_OXA-23_-like gene variants with *bla*_KPC_ and *bla*_VIM_ with our data was not possible.

Another insufficiency of the Check Direct CPE kit is that it was not able to detect *bla*_OXA-40_-like and *bla*_OXA-58_ due to the fact that the assay was designed for the detection of OXA-48-like. In outbreak situations this may increasingly become a problem, especially in outbreaks with *A. baumannii* isolates, the detection for such emerging subtypes in clinical specimens is mandatory for preventive and control measures in hospital setting. Fournier [17] reviewed a large number of numerous outbreaks caused by *A. baumannii* occurred in France and in the United States. Many of the outbreak strains have been found at intensive care units. Kohlenberg et al. [18] reported an outbreak of carbapenem-resistant *A. baumannii* carrying the carbapenemase gene OXA-23-like in a German university medical centre and Rolain et al. [19] reported this for Qatar during the period of 2011–2012.

In brief, the simultaneous identification of *bla*_OXA_-like gene variants needs to be improved. However, our in-house assay displays a sensitivity rate of 100% for the detection of *bla*_OXA-23_-like. With our in-house RT-PCR assay we were not able to differentiate the subtypes of the individual gene variants. Considering the high number of the analysed isolates positive for *bla*_OXA-23_-like and *bla*_OXA-48_-like variants a stepwise diagnostic workflow with melting-curve analysis was performed to rather differentiate the most prevalent OXA subtypes in this study. The peak between 80.6 and 80.9 °C was observed for OXA-23-like and between 83.2 and 83.3 °C for OXA-48-like. This assay is advantageous for the epidemiological surveillance for outbreaks with isolates carrying carbapenemase *bla*_OXA_ gene variants and might be extended for other carbapenemase gene variants. Roth et al. [6] reported the differentiation and identification of *bla*_KPC-2_ and *bla*_KPC-3_ genes with high-resolution melting analysis with 100% specificity and sensitivity for the detection of *bla*_KPC_.

Regarding the importance of identification and antimicrobial susceptibility testing from positive blood-cultures due to septicaemia and septic shock [20] carbapenemase-producers were additionally identified directly from positive blood-culture isolates using the in-house assay. In brief, all investigated *bla*_VIM_ and *bla*_NDM_ isolates were identified by the in-house assay. In case of isolates carrying *bla*_KPC_ genes one (7.1%) out of 14 could not be detected. The bacterial pellet of this isolate was bloody after the second centrifugation step and not whitish as was observed for the other bacterial pellets, which might be the cause for the unsuccessful detection. For isolates containing *bla*_OXA_ gene variants the identification directly from positive blood vials could be observed for *bla*_OXA-23_-like and *bla*_OXA-48_-like (OXA-48 and -162). One *bla*_OXA-181_ (*bla*_OXA-48_-like) isolate could not be detected which is consistent with the PCR results from solid culture media. The amplification of one *bla*_OXA-232_ showed weaker PCR product. It should be emphasized that we used negative blood-culture bottles spiked with bacterial isolates to investigate the performance of the in-house RT-PCR. Noteworthy, whereas the phenotypic assays, e.g. MHT, are eligible to detect the carbapenemase activity irrespective of the carbapenemase encoding gene sequence the RT-PCR based assay can only detect known carbapenemase encoding genes and the number of carbapenemase encoding genes and allelic variants thereof is expanding rapidly.

We would like to address a few limitations of our study, e.g. the low number and diversity of the gram-negative bacterial isolates producing VIM subtypes and OXA-48-like enzymes—in contrast to OXA-23-like—and thus representing only one or few isolates of particular subtype. Based on our findings, we plan to conduct a longitudinal surveillance study to further elucidate the epidemiology of carbapenem non-susceptible gram-negative bacteria in our region.

In conclusion, we have demonstrated that the in-house multiplex RT-PCR was a successful tool for the identification of carbapenemase gene variants directly from different solid culture isolates as well as from positive blood-culture vials. The assay is easy to perform, interpret and allows distinction between *bla*_OXA-23_ and *bla*_OXA-48_ variants, e.g. for outbreak analysis. Our in-house RT-PCR assay is an easy and rapid method for the detection of carbapenem resistance genes as compared to the routine microbiological diagnostic methods which need 2–3 days. Such same day analysis is crucial in hospital setting to rather implement the infection control and prevention measures, and moreover to administer the appropriate antimicrobial therapy in severe infections.
